# Growth of HIV-exposed uninfected, compared with HIV-unexposed, Zambian children: a longitudinal analysis from infancy to school age

**DOI:** 10.1186/s12887-017-0828-6

**Published:** 2017-03-16

**Authors:** Anna Rosala-Hallas, Jonathan W. Bartlett, Suzanne Filteau

**Affiliations:** 1 Department of Biostatistics, University of Liverpool, Liverpool, UK; 2 Statistical Innovation Group, AstraZaneca, Cambridge, UK; 3 Department of Population Health, Faculty of Epidemiology and Population Health, London School of Hygiene & Tropical Medicine, Keppel Street, London, UK

**Keywords:** HIV-exposed uninfected, Child, Growth, Longitudinal

## Abstract

**Background:**

Early growth of HIV-exposed, uninfected (HEU) children is poorer than that of their HIV-unexposed, uninfected (HUU) counterparts but there is little longitudinal or longer term information about the growth effects of early HIV exposure.

**Methods:**

We performed a longitudinal analysis to compare growth of HEU and HUU infants and children using data from two cohort studies in Lusaka, Zambia. Initially 207 HUU and 200 HEU infants from the Breastfeeding and Postpartum Health (BFPH) study and 580 HUU and 165 HEU from the Chilenje Infant Growth, Nutrition and Infection Study (CIGNIS) had anthropometric measurements taken during infancy and again when school-aged, at which time 66 BFPH children and 326 CIGNIS children were available. We analysed the data from the two cohorts separately using linear mixed models. Linear regression models were used as a secondary analysis at the later time points, adjusting for breastfeeding duration. We explored when the main group differences in growth emerged in order to estimate the largest ‘effect periods’.

**Results:**

After adjusting for socioeconomic status and maternal education, HEU children had lower weight-for-age, length-for-age and BMI-for-age Z-scores during early growth and these differences still existed when children were school-aged. Exposure group differences changed most between 1 and 6 weeks and between 18 months and ~7.5 years.

**Conclusions:**

HEU children have poorer early growth than HUU children which persists into later growth. Interventions to improve growth of HEU children need to target pregnant women and infants.

**Electronic supplementary material:**

The online version of this article (doi:10.1186/s12887-017-0828-6) contains supplementary material, which is available to authorized users.

## Background

Africa contains the majority of the world’s Human Immunodeficiency Virus (HIV)-infected people. UNAIDS estimated that there were 27,000 deaths from Acquired Immunodeficiency Syndrome (AIDS) in Zambia, and over 1 million people living with HIV in 2013 [1]. There has been a big drive to eliminate mother-to-child HIV transmission and to do so by 2015 was one of the targets of the 2011 United Nations Zambia Country Progress Report [2]. As a result of public health interventions, rates of mother-to-child HIV transmission have been falling and more children are being born HIV-exposed yet uninfected (HEU).

There has been some research into the health and growth of HEU children from different parts of the world [3, 4]. However, most data from Africa, the centre of the HIV epidemic, is restricted to infancy and early childhood [5–10] or lacks appropriate non-HIV-exposed controls [11]. In spite of these data limitations, as well as the varying exposure to antiretroviral drugs in these studies, there is evidence of both linear and ponderal growth faltering in HEU children. The aim of this analysis was to determine whether the early and later growth, measured as weight-for-age, height-for-age and body mass index (BMI)-for-age Z-scores, of HIV-unexposed-uninfected (HUU) and HEU children differ. The analysis also aimed to estimate the ‘effect periods’, i.e. those periods when the difference between the two exposure groups changed the most, in order to attempt to determine whether effects occur solely *in utero,* in infancy, or the effect which occurs *in utero* or in infancy then worsens or improves as the child grows. We controlled for socioeconomic factors in order to estimate whether any differences were more likely biological or social.

## Methods

### Participants

The data used for the analysis came from two cohorts of children from Lusaka, Zambia. The Breastfeeding and Postpartum Health Study (BFPH) [6, 12] was conducted from June 2001 to July 2003. The HIV status of all mothers was known through antenatal antibody testing at the local government clinic. BFPH infants were not HIV-tested in the original study since they were followed to only 16 weeks of age at a time when PCR testing was unavailable at the site; we did not have ethical approval to test for HIV at later time points so have HIV status information only for those who happened to be tested at local clinics, likely because they were ill. At the time of the study the only antiretroviral regimen available for prevention of mother-to-child transmission (PMTCT) in the area was perinatal nevirapine to both mother and infant. The median duration of exclusive breast feeding was 6 weeks for HIV-infected women and 9 weeks for HIV-uninfected women [13] and the median duration of any breast feeding was 17 months and 18 months for these groups, respectively, for children who remained HEU (unpublished). In a previous analysis, early growth of infants from 218 HIV-infected and 211 HIV-uninfected mothers was compared at birth, 6 and 16 weeks [6]. HIV-exposed infants had lower weight-for-age and length-for-age Z-scores at each time of measurement, controlling for gestational age, compared to HUU infants. At a median age of 2.7 years a subset of 205 children (107 HEU, 98 HUU) were followed up and measurements of weights and lengths were taken [6]. A further follow-up of 66 children, median age 11.6 years, took place (33 HUU, 33 HEU) in May 2014 [14]. Further details of follow-up study methods have been described previously.

The Chilenje Infant Growth, Nutrition and Infection Study (CIGNIS) was a double-blind randomised controlled trial conducted from October 2005 to July 2009 [15]. 811 infants were enrolled aged 6 months and randomised to one of two complementary foods, differing in micronutrient content, for 12 months. Maternal and child HIV status was assessed by antibody testing in the local clinic; women were mostly tested during antenatal care and children were tested at 18 months. Anthropometric measurements were taken at 6, 9, 12, 15 and 18 months. At the time of the study perinatal nevirapine was the local regimen for PMTCT. Antiretroviral therapy (ART) was available only for adults with CD4 count < 200 cells/μL until towards the end of the study when the cut-off was changed to < 350 cells/μL; few of the CIGNIS children’s mothers were on any ART. HIV-infected mothers were less likely to initiate breast feeding and stopped earlier compared to uninfected mothers [16]. A random effects regression analysis (controlling for treatment group, visit, socioeconomic status, maternal education, current breast feeding and sex), which included only children who had been followed up for the complete 18 months, found that HEU children had lower mean length-for-age and weight-for-age Z-scores than HUU children [17]. For the present analyses, HIV-infected children and children whose mother’s HIV status was unknown were excluded. Hence, weights and heights for 745 children (580 HUU, 165 HEU) were included in analyses; birthweights were also available for 737 children. A subset of the children (247 HUU, 79 HEU) was followed up when the children were at a median age of 7.5 years, in May 2014 [14].

At the later time points in both studies only a subset of children originally recruited were followed-up due to a lack of substantial funding. Mothers previously HIV-negative were not retested for HIV since we were interested in children’s exposure in utero and in infancy.

### Anthropometry

Anthropometric measurements for both studies and all time points were conducted or supervised by the same research nurse. Standard methods were used [18] and measurements made in duplicate (BFPH) or triplicate (CIGNIS and follow-up). Training and monitoring of staff was by assessment of the technical error of measurement [19].

### Statistical analysis

All analyses were carried out in Stata v13. Separately in each study, a linear mixed model was used to estimate the differences in anthropometric Z-scores (based on the WHO 2006/2007 growth standards) [20] between HUU and HEU children. In the BFPH cohort weight-for-age Z-scores could not be compared at the later visit since WHO provides weight-for-age Z-score references for children only up to the age of 10 and all but one child were 10 years or over.

We assumed missing data were missing at random (MAR), which essentially means that drop out at a given follow-up time is independent of outcome at that time, after adjustment for the previous measures of outcome (and other covariates, if adjusted for) [21]. The outcomes were weight-for-age, length/height-for-age and BMI-for-age Z-scores. In order to give an estimate of the difference at each time point, these models included time as a categorical variable with an interaction between time and exposure group. Within-child residuals were modelled with an unstructured covariance matrix. These models account for correlations between repeated measurements of the same child over time and also include all available data, i.e. if a child had missing data at one of the visits they were not discarded from the analysis and the available data from previous visits was included resulting in more precise estimates in comparison to complete case analysis.

Maternal education and socioeconomic status are known to be associated with poor growth of children in this community [5]; hence both cohorts were adjusted according to information collected as part of each study. The Z-scores already control for age and sex so these factors were not further controlled for. The models estimating differences in the BFPH cohort were adjusted for maternal education (primary, secondary, tertiary) and housing density (low, middle, high) as housing density was the closest available indication of socioeconomic status, both measured at recruitment. The CIGNIS models were adjusted for maternal education (primary or less, secondary, college/university) and socioeconomic group, based on an asset index score (low, middle, high), both measured at recruitment. We did not adjust for treatment group from the CIGNIS trial when children were 6–18 months since it had limited effect even in infancy and none by the time children were school-aged.

Linear regression models, using only available data at each time point, were used as a secondary analysis for the later time points (~2.7, ~7.5 and ~11.6 years) also adjusting for breast feeding duration in order to estimate direct effects of HIV exposure not mediated via breast feeding. For BFPH this was categorised by <18 months, 18+ months since this was the median for all children. For CIGNIS, breast feeding duration was categorised by <12 months, 12+ months as a compromise since earlier papers used 6 months as a cut-off for HEU children and 18 months for HUU children. Note that median duration of breast feeding differed between the two cohorts because infants were born when different HIV and infant feeding recommendations were in place.

The linear mixed models were also used to estimate ‘effect periods’. The changes in differences of Z-scores between HEU and HUU children from each time point to its preceding time point were calculated. For instance, the difference in weight-for-age Z-scores at birth was subtracted from the difference in weight-for-age Z-scores at 6 months to estimate by how much the difference between HUU and HEU infants changed from birth to 6 months. P-values from Wald tests and 95% confidence intervals were calculated.

## Results

**Fig. 1:**
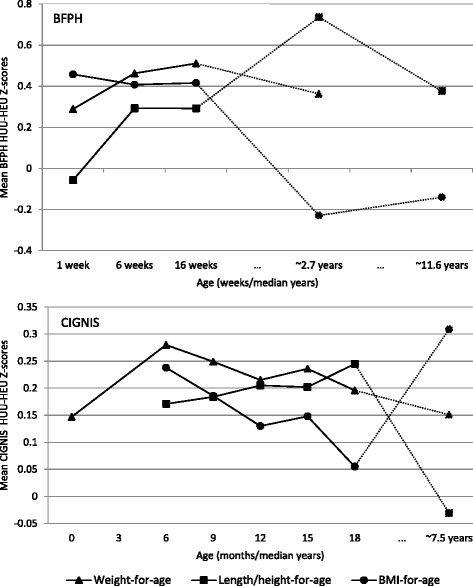
Estimated BFPH and CIGNIS adjusted^a^ differences in mean Z-scores between exposure groups given by the linear mixed models. ^a^Adjusted for maternal education (primary, secondary, tertiary) and housing density (high, medium, low) for BFPH and for maternal education (primary or less, secondary, tertiary) and socioeconomic group (low, middle, high) for CIGNIS

Table 1 provides descriptive data for both cohorts and Table 2 shows the number of children participating at each time point in the two studies. With the exception of week 1 length in the BFPH cohort, at each visit point estimates for anthropometry suggested that HEU children in both cohorts were shorter and lighter than HUU children (Table 2). For the BFPH cohort, after adjusting for maternal education and housing density, there was evidence at the 5% level that weight-for-age Z-scores were lower in HEU children compared with HUU children (Fig. 1 and Table 3) at all time points. There was evidence of borderline differences in length/height-for-age Z-scores at 6 weeks and at all subsequent time points. Up to 16 weeks there was strong evidence of differences in BMI-for-age Z-scores with HEU children estimated to have ~0.4 lower Z-scores than HUU children. There was no statistically significant evidence of differences at the later time points. A secondary analysis adjusting for breast-feeding duration at the later time points gave more conservative estimates for weight-for-age and height-for-age differences suggesting that some of the differences between the two groups are due to differences in breast-feeding duration (Additional file 1: Table S1). There was borderline difference in height-for-age at ~2.7 years but no evidence of other differences between the two groups of BFPH children.

**Table 1:** Baseline demographic summary statistics

		HIV-unexposed	HIV-exposed-uninfected
Breast Feeding and Postpartum Health (BFPH) study			
N		207 (50.9)	200 (49.1)
Sex (N % female)	-	107 (51.7)	106 (53.0)
Housing density (N %)	High	49 (22.6)	49 (23.2)
	Middle	139 (64.1)	140 (66.4)
	Low	29 (13.4)	22 (10.4)
Maternal Education (N %)	Primary	34 (15.7)	35 (16.6)
	Secondary	126 (58.1)	118 (55.9)
	Tertiary	57 (26.3)	58 (27.5)
Breast-fed (N %)	<18 months	32 (31.1)	52 (54.2)
	18+ months	71 (68.9)	44 (45.8)
Chilenje Infant Growth, Nutrition and Infection Study (CIGNIS)			
N		580 (77.9)	165 (22.1)
Sex (N % female)	-	302 (52.1)	91 (55.2)
Socioeconomic group^a^ (N %)	Low	179 (30.9)	67 (40.6)
	Middle	227 (39.1)	57 (34.6)
	High	174 (30.0)	41 (24.9)
Maternal Education (N %)	Primary or less	174 (30.0)	66 (40.0)
	Secondary	225 (38.8)	60 (36.4)
	College/university	181 (31.2)	39 (23.6)
Breast-fed (N %)	<12 months	150 (25.9)	138 (83.6)
	12+ months	430 (74.1)	27 (16.4)

^a^Based on an asset index score

**Table 2:** Numbers available, mean (SD) weights and lengths/heights for available data^a^ by maternal HIV exposure

Breast Feeding and Postpartum Health (BFPH) study				
Age (weeks/median years)	Weight (kg) - N, mean (SD)		Length/height (cm) - N, mean (SD)	
	HUU	HEU	HUU	HEU
1 week	207	200	174	171
	3.0 (0.4)	2.9 (0.5)	49.4 (2.6)	49.6 (2.6)
6 weeks	178	173	178	174
	5.1 (0.6)	4.8 (0.7)	55.0 (2.8)	54.4 (2.9)
16 weeks	175	168	174	169
	6.8 (0.8)	6.5 (1.0)	61.9 (2.6)	61.2 (3.4)
~2.7 years	107	98	107	98
	13.3 (2.1)	12.8 (2.4)	92.1 (8.3)	89.2 (7.9)
~11.6 years	33	33	33	33
	38.5 (10.1)	37.2 (8.9)	145.3 (7.9)	143.2 (8.7)
Chilenje Infant Growth, Nutrition and Infection Study (CIGNIS)				
Age (months/median years)	Weight (kg) - N, mean (SD)		Length/height (cm) - N, mean (SD)	
	HUU	HEU	HUU	HEU
Birth^b^	574	163	-	-
	3.1 (0.5)	3.0 (0.5)		
6 months	580	165	580	165
	7.4 (1.1)	7.1 (1.1)	65.0 (2.5)	64.3 (2.4)
9 months	506	152	506	152
	8.3 (1.2)	8.0 (1.2)	69.4 (2.6)	68.7 (2.8)
12 months	470	152	469	151
	9.0 (1.3)	8.7 (1.4)	73.0 (2.8)	72.3 (2.8)
15 months	446	144	445	142
	9.6 (1.4)	9.2 (1.4)	76.1 (2.9)	75.1 (3.2)
18 months	446	141	445	141
	10.2 (1.5)	9.8 (1.5)	78.8 (3.2)	77.6 (3.4)
~7.5 years	247	79	247	79
	23.8 (5.4)	22.5 (3.8)	122.4 (6.6)	122.0 (6.5)

*Abbreviations*: *HEU* HIV-exposed, uninfected, *HUU* HIV-unexposed, uninfected

^a^The majority of loss to follow-ups were inability to trace at later follow-ups for funding and other reasons. 21 infants died during the original BFPH study and 12 during the original CIGNIS study. We have no information about later deaths. 18 infants in the CIGNIS study were HIV-positive and thus excluded from the analysis

^b^Birth weights of CIGNIS children were taken from Road-to-Health cards

**Table 3:** Difference^a^ in mean Z-score by maternal HIV status [95% CI] as estimated by the linear mixed models

Breast Feeding and Postpartum Health (BFPH) study						
Age (weeks/median years)	Unadjusted			Adjusted^b^		
	WAZ	L/HAZ	BAZ	WAZ	L/HAZ	BAZ
1 week	0.28 0.[09, 0.47] *P* < 0.01	−0.07 [−0.34, 0.21] *P* = 0.64	0.44 [0.17, 0.72] *P* < 0.01	0.29 [0.10, 0.48] *P* < 0.01	−0.06 [−0.33, 0.22] *P* = 0.68	0.46 [0.19, 0.73] *P* < 0.01
6 weeks	0.47 [0.25, 0.69] *P* < 0.01	0.31 [0.01, 0.60] *P* = 0.04	0.42 [0.15, 0.69] *P* < 0.01	0.46 [0.25, 0.68] *P* < 0.01	0.29 [0.00, 0.59] *P* = 0.05	0.41 [0.14, 0.68] *P* < 0.01
16 weeks	0.52 [0.28, 0.75] *P* < 0.01	0.31 [0.02, 0.61] *P* = 0.04	0.42 [0.16, 0.69] *P* < 0.01	0.51 [0.27, 0.75] *P* < 0.01	0.29 [0.00, 0.59] *P* = 0.05	0.42 [0.15, 0.68] *P* < 0.01
~2.7 years	0.38 [0.02, 0.73] *P* = 0.04	0.76 [0.21, 1.32] *P* = 0.01	−0.23 [−0.77, 0.31] *P* = 0.41	0.36 [0.01, 0.71] *P* = 0.04	0.74 [0.18, 1.29] *P* < 0.01	−0.23 [−0.77, 0.31] *P* = 0.41
~11.6 years	-	0.32 [−0.10, 0.75] *P* = 0.14	−0.11 [−0.62, 0.40] *P* = 0.67	-	0.38 [−0.02, 0.77] *P* = 0.06	−0.14 [−0.65, 0.37] *P* = 0.59
Chilenje Infant Growth, Nutrition and Infection Study (CIGNIS)						
Age (months/median years)	Unadjusted			Adjusted^c^		
	WAZ	L/HAZ	BAZ	WAZ	L/HAZ	BAZ
Birth	0.19 [0.00, 0.38] *P* = 0.06	-	-	0.15 [−0.05, 0.34] *P* = 0.14	-	-
6 months	0.35 [0.14, 0.56] *P* < 0.01	0.24 [0.06, 0.42] *P* = 0.01	0.28 [0.07, 0.48] *P* < 0.01	0.28 [0.07, 0.49] *P* < 0.01	0.17 [0.00, 0.35] *P* = 0.06	0.24 [0.03, 0.44] *P* = 0.02
9 months	0.31 [0.10, 0.52] *P* < 0.01	0.26 [0.07, 0.45] *P* < 0.01	0.23 [0.02, 0.43] *P* = 0.03	0.25 [0.04, 0.45] *P* = 0.02	0.18 [0.00, 0.37] *P* = 0.05	0.19 [−0.01, 0.39] *P* = 0.07
12 months	0.32 [0.11, 0.53] *P* < 0.01	0.29 [0.10, 0.48] *P* < 0.01	0.20 [−0.01, 0.41] *P* = 0.06	0.22 [0.01, 0.42] *P* = 0.04	0.20 [0.02, 0.39] *P* = 0.03	0.13 [−0.07, 0.33] *P* = 0.21
15 months	0.36 [0.15, 0.57] *P* < 0.01	0.31 [0.12, 0.51] *P* < 0.01	0.23 [0.03, 0.42] *P* = 0.02	0.24 [0.03, 0.44] *P* = 0.02	0.20 [0.02, 0.39] *P* = 0.03	0.15 [−0.04, 0.34] *P* = 0.13
18 months	0.32 [0.11, 0.53] *P* < 0.01	0.39 [0.19, 0.59] *P* < 0.01	0.11 [−0.08, 0.30] *P* = 0.27	0.20 [−0.01, 0.40] *P* = 0.06	0.24 [0.06, 0.43] *P* = 0.01	0.05 [−0.14, 0.25] *P* = 0.58
~7.5 years	0.27 [0.02, 0.52] *P* = 0.03	0.05 [−0.15, 0.25] *P* = 0.63	0.41 [0.13, 0.70] *P* < 0.01	0.15 [−0.09, 0.39] *P* = 0.22	−0.03 [−0.23, 0.16] *P* = 0.75	0.31 [0.03, 0.59] *P* = 0.03

*WAZ* weight-for-age Z-score, *L/HAZ* length/height-for-age Z-score, *BAZ* BMI-for-age Z-score, *HEU* HIV-exposed uninfected, *HUU* HIV-unexposed, uninfected

Sample sizes: BFPH - 1 week: WAZ - 207 HUU, 200 HEU; L/HAZ – 174 HUU, 171 HEU; BAZ – 174 HUU, 171 HEU; 6 weeks: WAZ – 178 HUU, 173 HEU; L/HAZ – 178 HUU, 174 HEU; BAZ – 178 HUU, 173 HEU; 16 weeks: WAZ – 175 HUU, 168 HEU; BAZ – 174 HUU, 168 HEU; ~2.7 years: WAZ – 107 HUU, 98 HEU; L/HAZ – 107 HUU, 98 HEU; BAZ – 107 HUU, 98 HEU; ~11.6 years: WAZ – 33 HUU, 33 HEU; L/HAZ – 33 HUU, 33 HEU, BAZ – 33 HUU, 33 HEU CIGNIS - Birth: WAZ – 574 HUU, 163 HEU; 6 months: WAZ – 580 HUU, 165 HEU; L/HAZ – 580 HUU, 165 HEU; BAZ – 580 HUU, 165 HEU; 9 months: WAZ – 506 HUU, 152 HEU; L/HAZ – 506 HUU, 152 HEU; BAZ – 506 HUU, 152 HEU; 12 months: WAZ - 470 HUU; 152 HEU; L/HAZ – 469 HUU; 151 HEU; BAZ – 469 HUU, 151 HEU; 15 months: WAZ – 446 HUU; 144 HEU; L/HAZ - 445 HUU; 142 HEU; BAZ – 445 HUU; 142 HEU; 18 months: WAZ – 446 HUU, 144 HEU; L/HAZ – 445 HUU, 141 HEU; BAZ – 445 HUU, 141 HEU; ~7.5 years: WAZ – 247 HUU; 79 HEU; L/HAZ – 247 HUU; 79 HEU; BAZ – 247 HUU, 79 HEU

^a^HUU mean Z-scores minus HEU mean Z-scores

^b^Adjusted for maternal education (primary, secondary, tertiary) and housing density (high, medium, low)

^c^Adjusted for maternal education (primary or less, secondary, tertiary) and socioeconomic group (low, middle, high)

For the CIGNIS children, in the adjusted analyses there was statistically significant evidence of differences in weight-for-age and length-for-age between the two groups of children at most time points from 6 months to 18 months but of differences in BMI-for-age only at 6 and 15 months and ~7.5 years (Fig. 1 and Table 3). By ~7.5 years HEU children had a 0.15 (95% CI [−0.09, 0.39] *p* = 0.22) lower weight-for-age Z-score compared with HUU children and an estimated 0.31 (95% CI [0.03, 0.59] *p* = 0.03) lower BMI-for-age. Height-for-age Z-scores did not differ between groups. When adjusting for breast-feeding duration at ~7.5 years in the secondary analysis, the point estimates of weight for age reduced very slightly (compared to those not adjusting for breast-feeding), while estimated differences in length-for-age and BMI-for-age increased somewhat. There was evidence of a difference (*p* = 0.02) in BMI-for-age Z-scores but not of differences in weight-for-age or height-for-age Z-scores, although confidence intervals were wide (Additional file 1: Table S1).

### Effect periods

Table 4 shows the estimated incremental changes over time in the exposure group differences in outcomes. In BFPH there was borderline statistically significant evidence of an increasing difference in weight-for-age (*p* = 0.06) and length-for-age (*p* = 0.03) Z-scores between 1 and 6 weeks suggesting that this could be a period where maternal HIV exposure has a large effect on growth. Differences in weight-for-age and length-for-age Z-scores then remained fairly constant between 6 and 16 weeks. Differences in BMI-for-age Z-scores remained fairly constant between 1 and 16 weeks but then there was evidence of a significant change (*p* = 0.03) between 16 weeks and ~2.7 years. Length/height-for-age exhibited a contrasting pattern between 16 weeks and ~2.7 years with the difference in length/height-for-age estimated to increase; however there was no evidence against the null of these changes in differences being zero. By ~11.6 years the change in group difference in BMI-for-age grew closer to zero and also decreased for length-for-age.

**Table 4:** Changes with time in differences^a^ of anthropometric Z-scores between the HUU and HEU children and infants

	Weight-for-age	Length/height-for-age	BMI-for-age
Breast Feeding and Postpartum Health (BFPH) study			
6–1 week	0.17 [0.01, 0.35] *P* = 0.06	0.35 [0.04, 0.66] *P* = 0.03	−0.05 [−0.39, 0.29] *P* = 0.77
16–6 weeks	0.05 [−0.13, 0.22] *P* = 0.59	0.00 [−0.27, 0.27] *P* = 0.99	0.01 [−0.27, 0.29] *P* = 0.95
~2.7 years-16 weeks	−0.15 [−0.51, 0.21] *P* = 0.41	0.44 [−0.13, 1.02] *P* = 0.13	−0.64 [−1.24, 0.05] *P* = 0.03
~11.6 - ~ 2.7 years	-	−0.36 [−0.95, 0.24] *P* = 0.24	0.09 [−0.64, 0.82] *P* = 0.81
Chilenje Infant Growth, Nutrition and Infection Study (CIGNIS)			
6 months-birth	0.13 [−0.08, 0.35] *P* = 0.22	-	-
9–6 months	0.03 [−0.12, 0.06] *P* = 0.50	0.01 [−0.08, 0.10] *P* = 0.78	0.05 [−0.17, 0.07] *P* = 0.39
12–9 months	−0.03 [−0.12, 0.05] *P* = 0.43	0.02 [−0.07, 0.11] *P* = 0.65	−0.06 [−0.17, 0.06] *P* = 0.35
15–12 months	0.02 [−0.05, 0.10] *P* = 0.58	0.00 [−0.08, 0.08] *P* = 0.96	0.02 [−0.09, 0.13] *P* = 0.74
18–15 months	0.04 [−0.11, 0.03] *P* = 0.25	0.04 [−0.04, 0.12] *P* = 0.30	−0.09 [−0.20, 0.01] 0.08
~7.5 years-18 months	−0.04 [−0.27, 0.18] *P* = 0.70	−0.28 [−0.46, −0.09] *P* < 0.01	0.25 [−0.02, 0.53] *P* = 0.07

*WAZ* weight-for-age Z-score, *L/HAZ* length/height-for-age Z-score, *BAZ* BMI-for-age Z-score, *HEU* HIV-exposed, uninfected, *HUU* HIV-unexposed, uninfected

Sample sizes: BFPH - 1 week: WAZ - 207 HUU, 200 HEU; L/HAZ – 174 HUU, 171 HEU; BAZ – 174 HUU, 171 HEU; 6 weeks: WAZ – 178 HUU, 173 HEU; L/HAZ – 178 HUU, 174 HEU; BAZ – 178 HUU, 173 HEU; 16 weeks: WAZ – 175 HUU, 168 HEU; BAZ – 174 HUU, 168 HEU; ~2.7 years: WAZ – 107 HUU, 98 HEU; L/HAZ – 107 HUU, 98 HEU; BAZ – 107 HUU, 98 HEU; ~11.6 years: WAZ – 33 HUU, 33 HEU; L/HAZ – 33 HUU, 33 HEU, BAZ – 33 HUU, 33 HEU CIGNIS - Birth: WAZ – 574 HUU, 163 HEU; 6 months: WAZ – 580 HUU, 165 HEU; L/HAZ – 580 HUU, 165 HEU; BAZ – 580 HUU, 165 HEU; 9 months: WAZ – 506 HUU, 152 HEU; L/HAZ – 506 HUU, 152 HEU; BAZ – 506 HUU, 152 HEU; 12 months: WAZ - 470 HUU; 152 HEU; L/HAZ – 469 HUU; 151 HEU; BAZ – 469 HUU, 151 HEU; 15 months: WAZ – 446 HUU; 144 HEU; L/HAZ - 445 HUU; 142 HEU; BAZ – 445 HUU; 142 HEU; 18 months: WAZ – 446 HUU, 144 HEU; L/HAZ – 445 HUU, 141 HEU; BAZ – 445 HUU, 141 HEU; ~7.5 years: WAZ – 247 HUU; 79 HEU; L/HAZ – 247 HUU; 79 HEU; BAZ – 247 HUU, 79 HEU

^a^Difference between HUU minus HEU at the later time point minus difference at the earlier time point, i.e. positive values indicate increasing differences

For CIGNIS children, differences in weight-for-age Z-scores remained fairly constant from birth up to ~7.5 years. Differences in length-for-age and BMI-for-age Z-scores remained fairly constant between 6 and 18 months. Between 18 months and ~7.5 years there was evidence for a decreased difference in length-for-age (*p* < 0.01) and an increased difference in BMI-for-age (*p* = 0.07).

## Discussion

The results indicate that the differences in growth between HEU and HUU occur mainly before birth or in early life with limited either worsening or catch-up in later childhood. Because both cohorts were from the same community and were studied by the same research team, it is worth considering their results together for a fuller picture of child growth differences, keeping in mind the large differences in breast-feeding practices of HIV-infected mothers in the BFPH and CIGNIS studies. In previous studies breast-feeding among HIV-exposed children has been associated with less faltering in early weight growth [22]. Although both HIV-infected and uninfected BFPH women breast-fed both exclusively and at all for similar lengths of time, the decreased point estimates of group differences when we controlled for breast-feeding duration supports this previous work. However, controlling for breast feeding in the CIGNIS study in which there were large differences in breast-feeding duration between HIV-infected and uninfected women had the opposite effect of increasing the point estimates of differences. Breast milk quality or quantity as well as breast-feeding duration may differ by maternal HIV status: in the BFPH study, the HIV-infected women had higher prevalence of subclinical mastitis, a condition which in dairy cows is associated with reduced milk production [12]. Although we did not measure breast milk production, reduced milk production by HIV-infected mothers is a possible cause of the increased weight and length growth faltering of their HEU infants between 1 and 6 weeks. Although we found similar early growth differentials between HEU and HUU children for length as for weight-for-age and BMI-for-age, other studies of HIV-exposed infants but lacking HUU controls found variable associations of infant feeding with length-for-age including no effect of breast feeding [23], reduced growth with formula feeding [22], and better length growth velocity with formula feeding [10]. It is likely that local socioeconomic conditions as well as trial designs which included providing formula account for these differences. In our study it is important to realise that the comparison group also growth faltered in infancy since Chilenje has a fairly high level of childhood stunting [24].

There are few published studies with which to compare our data from the older children. Weight-for-age differentials between HEU and HUU children showed relatively little change after 18 months. This is related to the opposing changes in BMI and height because of the definition, weight/height^2^, of BMI. In CIGNIS, between 18 months and ~7.5 years length-for-age Z-scores for the HEU children caught up with their unexposed peers but BMI differential worsened. In the BFPH cohort there was worsening length/height-for-age from 16 weeks to ~2.7 years and decreasing differential in BMI-for-age. This is different from a European cohort in which both weight and BMI appeared above the British growth standard mean by the time HEU children were school-aged [3]. It is possible that the timing of growth spurts differs between HEU and HUU children but this would require further research.

Previous reports of growth of the BFPH [6] and CIGNIS [5] children focussed on early growth and in the current paper we present an extension of this. Furthermore, growth was previously analysed in the CIGNIS cohort only for those who had complete data up to 18 months and in BFPH results were estimated using complete case analysis. Due to the nature of mixed models we have included all available data in our analysis, which leads to more precise estimates.

Both BFPH and CIGNIS cohorts were born at a time when ART availability was limited so the only drug used for PMTCT was perinatal nevirapine. It is possible that ART during pregnancy could reduce the *in utero* and infancy growth differentials. However, since there is evidence that some ART drugs during pregnancy are associated with *in utero* growth deficits [7], the growth differentials between HEU and HUU children might actually increase. In a European cohort, the increased availability of ART in the 1990s had little effect on the growth of HEU children [3]. Further research with African infants exposed to ART *in utero* is needed.

One limitation of the analysis was that there were no intermediate time points between 16 weeks, ~2.7 years and ~11.6 years and between 18 months and ~7.5 years which might have enabled effect periods to be defined more precisely. Another limitation of the study is the fact that only subsets of the original populations were followed-up at the later time points - these subsets, in addition to being fairly small, tended to be of a higher socioeconomic status (i.e. those owning a telephone) and for this reason socio-economic status was taken into consideration when making adjustments in the analyses. Our estimates could be biased however, if it were the case that those children whose outcomes were not available at the later time were systematically different, after taking into account baseline variables and early childhood outcome measures (the missing at random assumption). In addition, there could be residual, unmeasured confounding. Finally, for ethical reasons we did not retest previously HIV-negative children and relied on symptoms to determine if any were likely to be HIV-infected; there could thus have been some HIV-infected slow progressors among the HEU group. However, in a cross-sectional study of these children, a secondary analysis including only children confirmed HIV-negative found very similar results to the whole cohort [14].

## Conclusion

There is very limited literature on the growth of HEU children and much of what exists compares their growth to HIV-infected children [22, 25, 26] as opposed to using a HUU comparison group. We have been able to compare growth of HEU children with that of an HUU comparison group from the same population and of similar socioeconomic status. HEU children had lower weight-for-age, length-for-age and BMI-for-age Z-scores during early growth and these differences still existed when children were school-aged. This suggests that interventions to improve growth need to target pregnant women and infants. Further work into exploring the effect periods, including among children whose mothers had access to ART during pregnancy, would enable action to be taken in order to prevent these differences and ensure the good health of this growing population. Obviously additional efforts to decrease the number of women becoming HIV-infected are also required in order to reduce infant HIV exposure.

## Additional file

Additional file 1: Table S1. Difference in mean Z-scores at later time points by maternal HIV status [95% CI] as estimated by regression models adjusted for socioeconomic status, maternal education and breast-feeding duration. (PDF 236 kb)

## Abbreviations

BFPH: Breastfeeding and postpartum health study
BMI: Body mass index
CIGNIS: Chilenje growth nutrition and infection study
HEU: HIV-Exposed, uninfected
HUU: HIV-Unexposed, uninfected
PMTCT: Prevention of mother-to-child HIV transmission

## References

[1] UNAIDS (2014). Zambia HIV and AIDS estimates (2013) 2013.

[2] UNGASS. Zambia Country Report: Monitoring the Declaration of Commitment on HIV and AIDS and the Universal Access. Zambia: 2011. http://www.unaids.org/sites/default/files/country/documents/ZMB_narrative_report_2014.pdf. Accessed 17 Aug 2015.

[3] Newell ML, Borja MC, Peckham C, European Collaborative S (2003). Height, weight, and growth in children born to mothers with HIV-1 infection in Europe. Pediatrics.

[4] Isanaka S, Duggan C, Fawzi WW (2009). Patterns of postnatal growth in HIV-infected and HIV-exposed children. Nutr Rev.

[5] Filteau S, Baisley K, Chisenga, Kasonka L, Gibson R, CIGNIS Study Team (2011). Provision of micronutrient-fortified food from 6 months of age does not permit HIV-exposed, uninfected Zambian children to catch up in growth to HIV-unexposed children: a randomised controlled trial. J AIDS.

[6] Makasa M, Kasonka L, Chisenga M, Sinkala M, Chintu C, Tomkins A (2007). Early growth of infants of HIV-infected and uninfected Zambian women. Trop Med Int Health.

[7] Powis KM, Smeaton L, Ogwu A, Lockman S, Dryden-Peterson S, van Widenfelt E (2011). Effects of in utero antiretroviral exposure on longitudinal growth of HIV-exposed uninfected infants in Botswana. J Acquir Immune Defic Syndr.

[8] Muhangi L, Lule SA, Mpairwe H, Ndibazza J, Kizza M, Nampijja M (2013). Maternal HIV infection and other factors associated with growth outcomes of HIV-uninfected infants in Entebbe, Uganda. Public Health Nutr.

[9] Ramokolo V, Lombard C, Fadnes LT, Doherty T, Jackson DJ, Goga AE (2014). HIV infection, viral load, low birth weight, and nevirapine are independent influences on growth velocity in HIV-exposed South African infants. J Nutr.

[10] McGrath CJ, Nduati R, Richardson BA, Kristal AR, Mbori-Ngacha D, Farquhar C (2012). The prevalence of stunting is high in HIV-1-exposed uninfected infants in Kenya. J Nutr.

[11] Filteau S (2009). The HIV-exposed, uninfected African child. Trop Med Int Health.

[12] Kasonka L, Makasa M, Marshall T, Chisenga M, Sinkala M, Chintu C (2006). Risk factors for subclinical mastitis among HIV-infected and uninfected women in Lusaka, Zambia. Paediatr Perinat Epidemiol.

[13] Chisenga M, Kasonka L, Makasa M, Sinkala M, Chintu C, Kaseba C (2005). Factors affecting the duration of exclusive breastfeeding among HIV-infected and -uninfected women in Lusaka, Zambia. J Hum Lact.

[14] Nicholson L, Chisenga M, Siame J, Kasonka L, Filteau S (2015). Growth and health outcomes at school age in HIV-exposed, uninfected Zambian children: follow-up of two cohorts studied in infancy. BMC Pediatr.

[15] Chilenje Infant Growth Nutrition and Infection (CIGNIS) Study Team (2010). Micronutrient fortification to improve growth and health of maternally HIV-unexposed and exposed Zambian infants: a randomised controlled trial. PLoS One.

[16] Chisenga M, Siame J, Baisley K, Kasonka L, Filteau S (2011). Determinants of infant feeding choices by Zambian mothers: a mixed quantitative and qualitative study. Matern Child Nutr.

[17] Filteau S, Baisley K, Chisenga M, Kasonka L, Gibson RS, Team CS (2011). Provision of micronutrient-fortified food from 6 months of age does not permit HIV-exposed uninfected Zambian children to catch up in growth to HIV-unexposed children: a randomized controlled trial. J Acquir Immune Defic Syndr.

[18] Gibson RS (2005). Principles of Nutritional Assessment. 2nd Edition ed.

[19] Ulijaszek S, Kerr D (1999). Anthropometric measurement error and the assessment of nutritional status. Br J Nutr.

[20] Organization WH. Growth reference data for 5–19 years 2007 [17/06/2014]. Available from: http://www.who.int/growthref/en/. Accessed 17 Aug 2015.

[21] Beunckens C, Molenberghs G, Kenward MG (2005). Direct likelihood analysis versus simple forms of imputation for missing data in randomized clinical trials. Clin Trials.

[22] Venkatesh KK, Lurie MN, Triche EW, De Bruyn G, Harwell JI, McGarvey ST (2010). Growth of infants born to HIV-infected women in South Africa according to maternal and infant characteristics. Trop Med Int Health.

[23] Arpadi S, Fawzy A, Aldrovandi G, Kankasa C, Sinkala M, Mwiya M (2009). Growth faltering due to breastfeeding cessation in uninfected children born to HIV-infected mothers in Zambia. Am J Clin Nutr.

[24] Chilenje Infant Growth N, Infection Study T (2010). Micronutrient fortification to improve growth and health of maternally HIV-unexposed and exposed Zambian infants: a randomised controlled trial. PLoS One.

[25] Bobat R, Coovadia H, Moodley D, Coutsoudis A, Gouws E (2001). Growth in early childhood in a cohort of children born to HIV-1-infected women from Durban, South Africa. Ann Trop Paediatr.

[26] Owor M, Mwatha A, Donnell D, Musoke P, Mmiro F, Allen M (2013). Long-term follow-up of children in the HIVNET 012 perinatal HIV prevention trial: five-year growth and survival. J Acquir Immune Defic Syndr.

