# Supplementary material for: Growth of HIV-exposed uninfected, compared with HIV-unexposed, Zambian children: a longitudinal analysis from infancy to school age
Source: BMC Pediatr. 2017 Mar 16;17:80. doi: 10.1186/s12887-017-0828-6 (PMC5356250; doi:10.1186/s12887-017-0828-6)
Supplement: Additional file 1: Table S1. — Difference in mean Z-scores at later time points by maternal HIV status [95% CI] as estimated by regression models adjusted for socioeconomic status, maternal education and breast-feeding duration. (PDF 236 kb) [file 12887_2017_828_MOESM1_ESM.pdf]

Table S1: Difference[a] in mean Z-scores at later time points by maternal HIV status [95% CI] as estimated by regression models adjusted for socioeconomic status, maternal education and breast-feeding duration

	**Unadjusted**			**Adjusted[b]**		
	Weight-for-age	Height-for-age	BMI-for-age	Weight-for-age	Height-for-age	BMI-for-age
Age (years)	Breast Feeding and Postpartum Health (BFPH) study					
~2.7<br>N=205	0.24<br>[-0.13, 0.61]<br>P=0.20	0.69<br>[0.12, 1.27]<br>P=0.02	-0.24<br>[-0.79, 0.31]<br>P=0.39	0.12<br>[-0.26, 0.51]<br>P=0.52	0.60<br>[-0.02, 1.21]<br>P=0.06	-0.33<br>[-0.92, 0.26]<br>P=0.27
~11.6<br>N=66	-	0.22<br>[-0.28, 0.71]<br>P=0.38	-0.11<br>[-0.69, 0.47]<br>P=0.70	-	0.19<br>[-0.37, 0.75]<br>P=0.51	-0.07<br>[-0.79, 0.65]<br>P=0.84
	Chilenje Infant Growth, Nutrition and Infection Study (CIGNIS)					
~7.5<br>N=326	0.27<br>[-0.02, 0.56]<br>P=0.06	0.01<br>[-0.23, 0.24]<br>P=0.96	0.40<br>[0.09, 0.71]<br>P=0.01	0.24<br>[-0.14, 0.62]<br>P=0.21	0.14<br>[-0.45, 0.17]<br>P=0.38	0.51<br>[0.09, 0.92]<br>P=0.02

[a]HUU mean Z-scores minus HEU mean Z-scores.
[b]BFPH adjusted for maternal education (primary, secondary, tertiary), housing density (high, medium, low) and breastfeeding duration (<18 months, at least 18 months) and CIGNIS adjusted for maternal education (primary or less, secondary, tertiary), socioeconomic group (low, middle, high) and breastfeeding duration (<12 months, at least 12 months).
